# Supplementary figures and images for: Human iPSC-derived myelinating organoids and globoid cells to study Krabbe disease
Source: PLoS One. 2024 Dec 5;19(12):e0314858. doi: 10.1371/journal.pone.0314858 (PMC11620608; doi:10.1371/journal.pone.0314858)

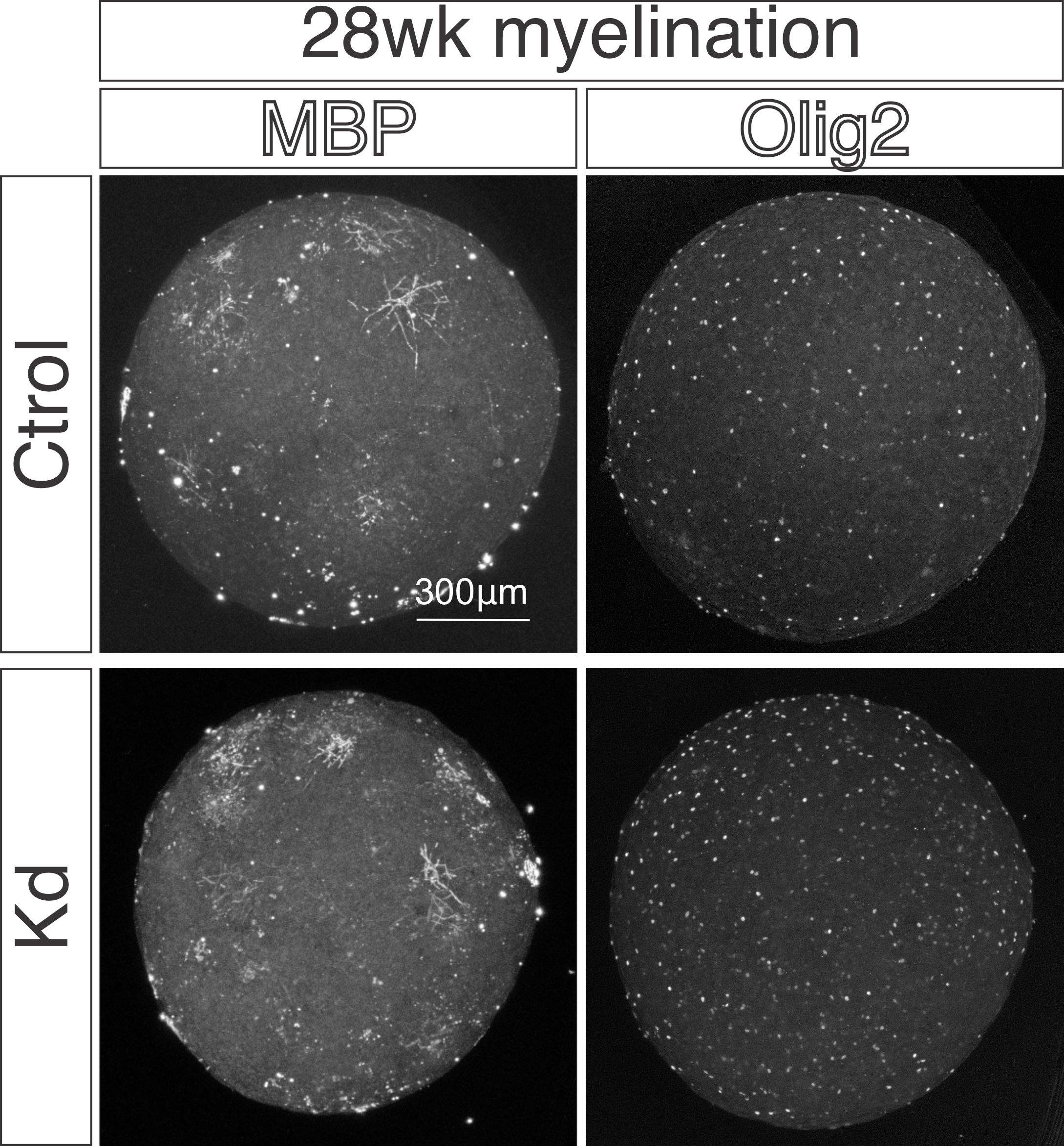

Supplement: S1 Fig — Immunohistochemistry for MBP and Olig2 of Ctrol and Kd Organoids at 28 weeks of myelination. (TIF) [file pone.0314858.s001.tif]

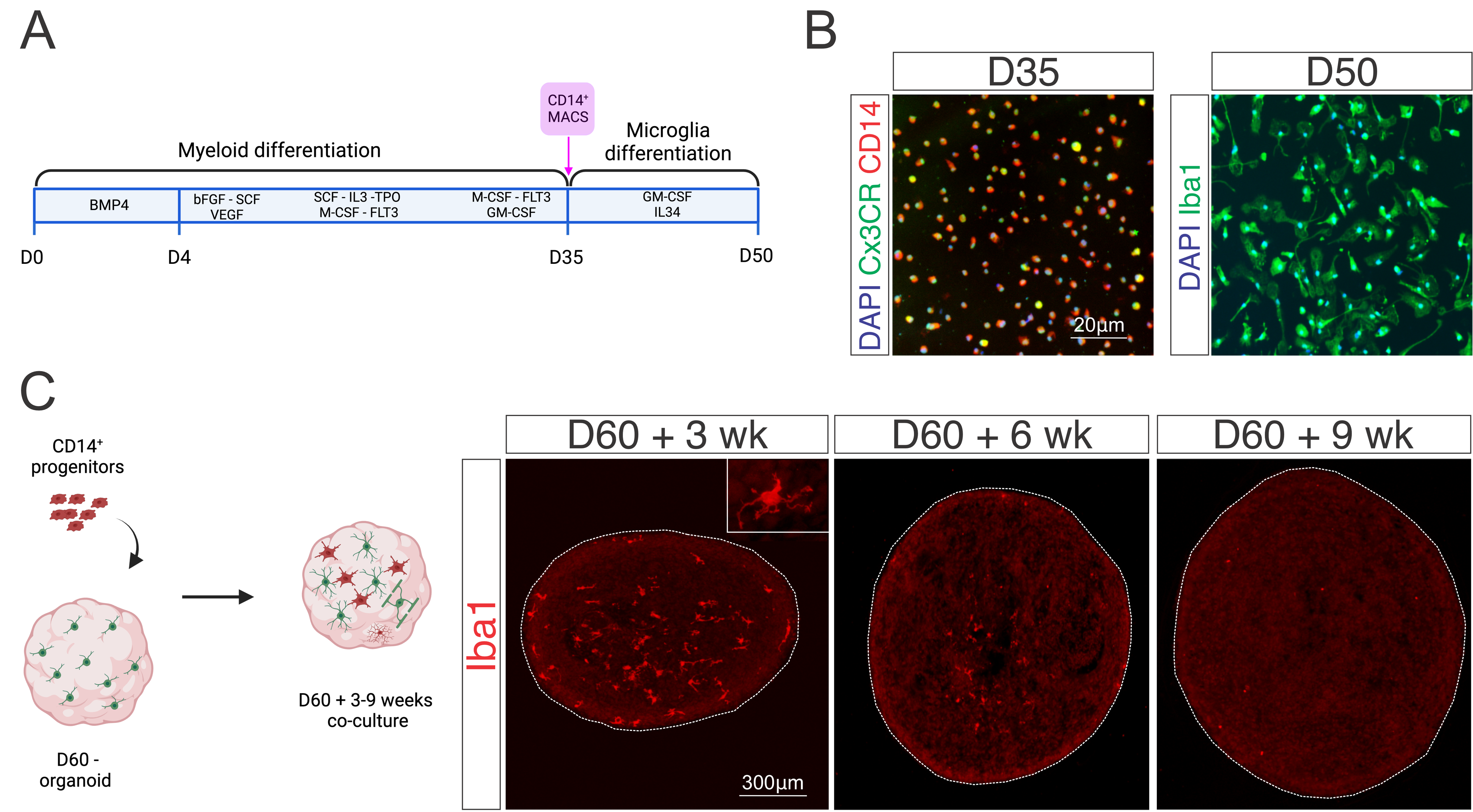

Supplement: S2 Fig — (A) Graphical abstract of the protocol used to produce iPSC derived microglia. (B) Immunohistochemistry for Cx3R1+/CD14+ microglial progenitors immediately after isolation (D35) and Iba1+ microglia after 14 days of differentiation (D50). (C) Microglial progenitors’ (D35) engraftment into D60 organoids. Immunohistochemistry for Iba1+ microglia engrafted into organoids 3, 6 and 9 weeks after engraftment. (TIF) [file pone.0314858.s002.tif]

Figure 3. Blots

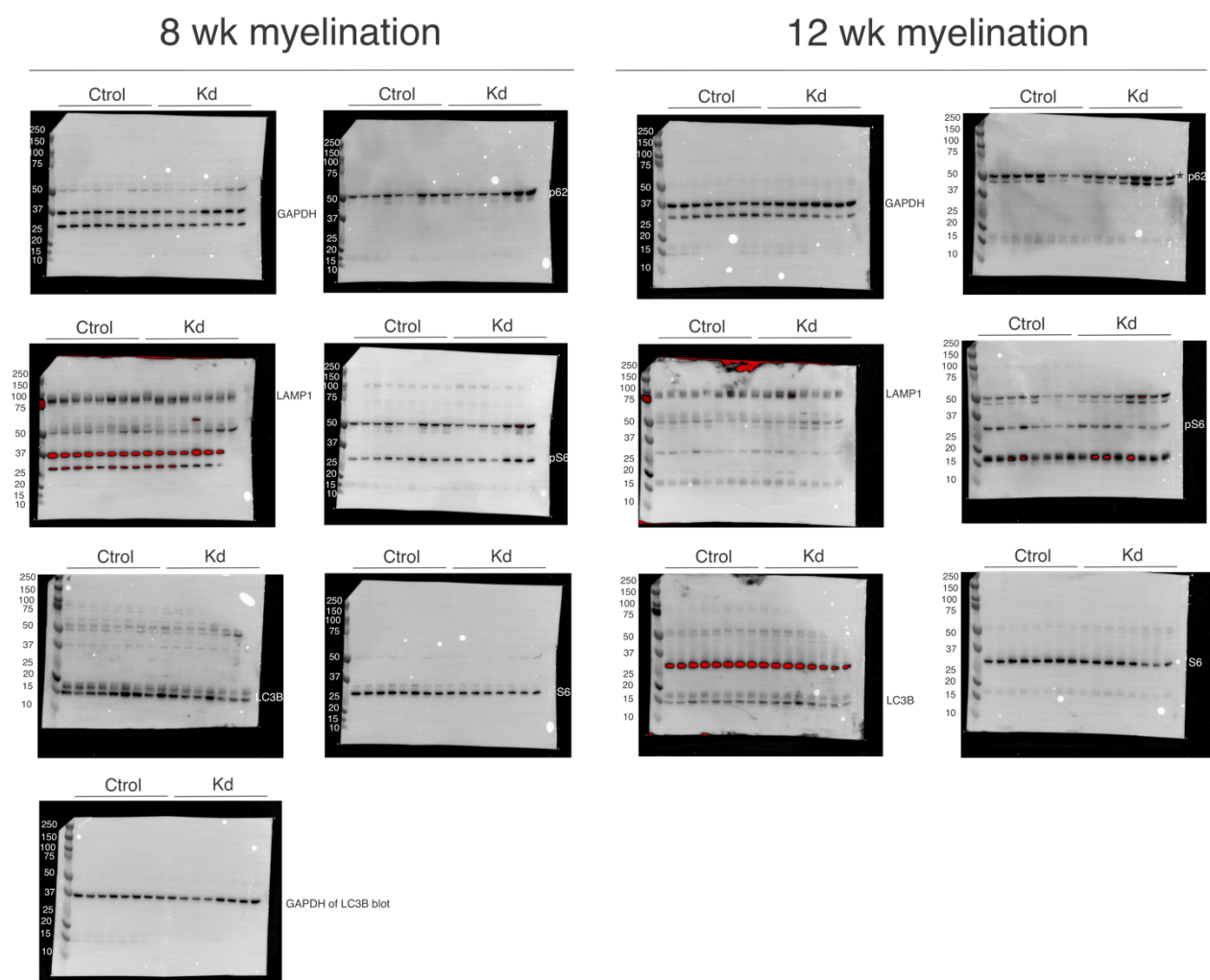

Figure 4. Blots

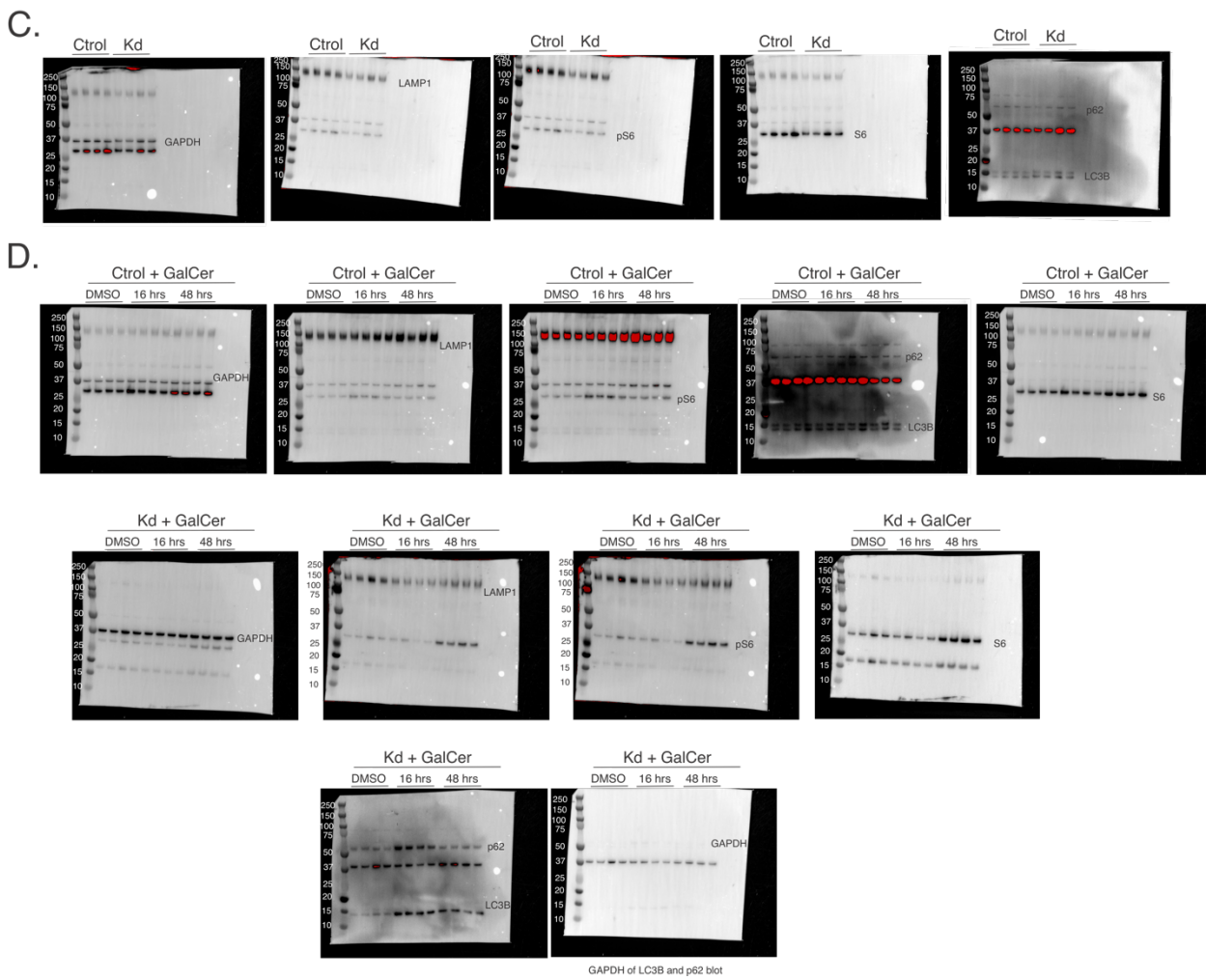

Supplement: S1 Raw image — (PDF) [file pone.0314858.s004.pdf]
